# Disaster exposure and women’s mental and general health: evidence from longitudinal data

**DOI:** 10.1080/16549716.2026.2752483

**Published:** 2026-10-27

**Authors:** Minnat Seema, Clifford Afoakwah, Joshua Byrnes

**Affiliations:** a Centre for Applied Health Economics, Griffith University, Brisbane, Queensland, Australia; b Australian Centre for Health Services Innovation, School of Public Health and Social Work, Queensland University of Technology, Brisbane, Queensland, Australia

**Keywords:** Disaster exposure, mental health, psychological distress, longitudinal data, fixed-effects models

## Abstract

**Background:**

Disasters can have important consequences for health, yet much evidence is cross-sectional and cannot account for time-invariant individual heterogeneity.

**Objective(s):**

To examine the association between reported disaster exposure and women’s mental health, psychological distress, and general health using longitudinal Australian data.

**Methods:**

Data from women aged ≥ 15 years participating in Waves 9–22 of the Household, Income and Labour Dynamics in Australia Survey were analysed. Individual and survey-wave fixed-effects models examined within-person associations between disaster exposure and mental health (MHI-5), psychological distress (Kessler-10), and SF-36 General Health. Sensitivity analyses considered long-term illness and single exposure. Dynamic associations were examined using the Callaway–Sant’Anna multiple-period difference-in-differences estimator with formal pre-trend tests.

**Results:**

Reported disaster exposure was associated with modest declines in mental health (β = −0.86, 95% CI − 1.51 to −0.20) and general health (β = −0.83, 95% CI − 1.44 to −0.22). Psychological distress was higher but not statistically significant in the primary model (β = 0.33, 95% CI − 0.04 to 0.70). Sensitivity analyses generally supported the mental and general health findings. Event-study analyses showed no consistent evidence of persistent post-exposure deterioration; however, the MHI-5 joint pre-trend test indicated differential pre-exposure patterns, limiting causal interpretation.

**Conclusions:**

Reported disaster exposure was associated with modest within-person declines in women’s mental and general health, while evidence for psychological distress was less conclusive. The findings support consideration of women’s health in disaster preparedness and recovery planning.

## Paper Context

- **Main findings**: Reported disaster exposure was associated with modest declines in women’s mental and general health, while evidence for increased psychological distress was less conclusive.
- **Added knowledge**: This longitudinal study provides evidence of within-individual health changes associated with reported disaster exposure while accounting for time-invariant individual characteristics.
- **Global health impact for policy and action**: The findings support consideration of women’s mental and general health in disaster preparedness and recovery planning.

## Background

Disasters represent major population-level disruptions with potentially substantial and persistent consequences for health. A growing body of evidence indicates that disaster exposure is associated with poorer mental health, greater psychological distress, and poorer general health, with adverse associations potentially extending beyond the immediate post-disaster period [1–6]. From a health economics perspective, these health changes are important because disaster may have broader implications for labour supply, healthcare utilisation, household resources, productivity, and long-term wellbeing, although these downstream economic consequences are not directly examined in the present study [7,8].

Despite considerable research, much of the evidence on disaster-related health associations relies on cross-sectional or short-panel studies that cannot adequately distinguish changes associated with disaster exposure from pre-existing health differences or other unobserved individual characteristics [3,9]. This limitation is particularly important for mental health outcomes, which may be influenced by stable personal and socioeconomic characteristics. Longitudinal analyses that exploit within-person variation provide a stronger framework for examining disaster-related changes in health by controlling for time-invariant individual heterogeneity and common temporal shocks [10–12]. Recent Australian longitudinal evidence using HILDA has further demonstrated the importance of considering repeated disaster exposure when examining mental health trajectories [13]

Women represent an important population for examining disaster-related health vulnerability. Previous research has identified several pathways that may shape women’s experiences following disasters, including caregiving responsibilities, labour-market disruption, household and financial pressures, and unequal access to recovery resources [14–20]. Recent evidence from women affected by the 2023 Türkiye earthquake also demonstrated substantial psychological distress and highlighted the potential importance of injury, bereavement, loss of shelter, and other disaster-related circumstances in shaping women’s mental health [21]. These pathways may contribute to adverse psychological and broader health outcomes, although vulnerability is heterogeneous among women and may vary according to socioeconomic circumstances, age, health status, family responsibilities, location, housing security, and access to services. Recent evidence also highlights the importance of considering intersecting socioeconomic, geographic, and health-related vulnerabilities when examining mental health following natural hazards [22]. The present study therefore examines health changes within women rather than testing whether disaster-related associations differ between women and men.

This study contributes to the applied health economics literature by providing longitudinal evidence on the association between reported disaster exposure and women’s mental health, psychological distress, and general health. Using longitudinal panel data from the Household, Income and Labour Dynamics in Australia (HILDA) Survey, we employ individual fixed-effects models with survey wave fixed effects and an event-study framework to examine within-person changes in health associated with reported disaster exposure while accounting for time-invariant individual characteristics and common temporal shocks [23,24].

The three outcomes capture related but distinct dimensions of health. The MHI-5 captures mental health and emotional wellbeing, while the Kessler-10 measures symptoms of nonspecific psychological distress. The SF-36 General Health scale captures a broader perception of overall health that may encompass physical and psychological wellbeing [25,26]. Examining these outcomes jointly therefore allows us to assess whether reported disaster exposure is associated primarily with psychological wellbeing and distress or also with broader perceived health.

In addition, this study examines the temporal pattern of health changes before and after reported disaster exposure using an event-study framework, allowing assessment of pre-exposure patterns and whether associations emerge around the exposure period or continue over subsequent survey waves [21,22]. Recent longitudinal research has similarly highlighted the importance of examining longer-term trajectories of psychological adjustment following disaster exposure [6]. By providing longitudinal evidence on women’s health surrounding reported disaster exposure, the study may inform disaster preparedness, mental health service planning, and public health policy while recognising that the analysis does not directly evaluate specific policy interventions.

## Methods

### Study design and data

This longitudinal panel study examined within-individual associations between disaster exposure and women’s health using data from the HILDA Survey, a nationally representative annual household panel that has followed Australian residents since 2001. The analysis used survey waves 9–22, corresponding to 2009–2022, and the period for which the disaster-exposure measure was available.

The analytical sample comprised women aged 15 years and older with non-missing information on disaster exposure and the relevant health outcome. Women who did not provide a valid response to the disaster-exposure question were excluded from the relevant analysis. The panel was unbalanced, with participants contributing varying numbers of observations across survey waves. The analysis focused on women because previous evidence suggests that disaster-related health responses differ by gender.

### Outcome variables

Three health outcomes were examined: (1) mental health, measured using the five-item SF-36 Mental Health Index (MHI-5); (2) general health, measured using the five-item SF-36 General Health scale; and (3) psychological distress, measured using the 10-item Kessler Psychological Distress Scale (K10). The MHI-5 and SF-36 General Health measures were available annually, whereas the K10 measure was available in waves 9, 11, 13, 15, 17, 19, and 21, resulting in a smaller K10 analytical sample.

The HILDA-derived MHI-5 and SF-36 General Health scores were analysed on scales ranging from 0 to 100, with higher scores indicating better health. K10 item responses were combined to produce a score ranging from 10 to 50, with higher scores indicating greater psychological distress. The analysis used the HILDA-provided derived scores and did not undertake additional item-level imputation. Observations without a valid derived score, including negative survey codes and other invalid responses, were excluded from the relevant outcome analysis.

### Key explanatory variable: disaster exposure

Disaster exposure was measured using the HILDA variable *ledhm*, based on respondents’ responses to the item asking whether, during the previous 12 months, ‘a weather-related disaster (e.g. flood, bushfire, cyclone) damaged or destroyed your home.’ A time-varying binary indicator was constructed and coded as one in survey waves in which a respondent reported exposure and zero when the respondent reported no exposure. Non-response, refusal, and other invalid survey codes were treated as missing rather than being classified as non-exposure.

Disaster exposure was reported in the current survey wave with reference to the preceding 12 months, while the health outcomes were collected during the same survey wave. Therefore, the precise timing of the disaster relative to the health assessment could not be established. The exposure measure also could not distinguish multiple disaster events occurring between survey interviews.

### Time-varying covariates

Long-term illness was included as a time-varying covariate in a secondary sensitivity specification. It was coded as one when a respondent reported a long-term health condition, impairment, or disability that restricted everyday activities and had lasted, or was likely to last, for 6 months or more, and zero otherwise. Invalid responses were treated as missing. Because this information was collected during the same survey wave as disaster exposure and the health outcomes, long-term illness was treated as a sensitivity variable rather than a primary confounder.

Age was used to define the principal analytical sample of women aged 15 years and older but was not included as a separate covariate in the fixed-effects models. Within an annual panel, age changes systematically with survey wave and is therefore not separately identifiable from individual and survey-wave fixed effects. As a sensitivity analysis, all principal models were repeated after restricting the sample to women aged 18 years and older.

Supplementary fixed-effects specifications additionally examined geographic remoteness, educational attainment, and employment status as contextual and socioeconomic characteristics. Each characteristic was examined in a separate model that also included disaster exposure, long-term illness, individual fixed effects, and survey-wave fixed effects. These characteristics were excluded from the primary specification because they may be relatively stable over time or may change following disaster exposure or health shocks. They were therefore included only in supplementary specifications.

### Quantitative variables

Disaster exposure and long-term illness were analysed as binary time-varying variables. Mental health, self-rated general health, and psychological distress were analysed on their original continuous scales. Age was retained in years and used to define the age-based analytical samples and for descriptive purposes. No continuous variables were categorised in the primary analyses.

### Bias

Several strategies were used to minimise potential sources of bias. Individual fixed-effects models controlled for all observed and unobserved individual characteristics that remained constant over time. Survey wave fixed effects accounted for common temporal shocks affecting all participants in a given wave. A secondary specification included time-varying long-term illness to assess whether the estimates were sensitive to changes in chronic health status. Because long-term illness could potentially arise following disaster exposure and lie on the pathway between exposure and health, it was not included in the primary specification. Robust standard errors clustered at the individual level accounted for repeated observations within women.

Event-study models were used to assess changes in health before and after reported disaster exposure and to examine whether health outcomes exhibited differential pre-exposure trends. These analyses were restricted to women reporting disaster exposure in no more than one survey wave, reducing potential ambiguity arising from repeated reported exposures.

Nevertheless, disaster exposure was self-reported and may be subject to recall or reporting error. Residual confounding by unmeasured time-varying factors cannot be excluded. The estimated relationships are therefore interpreted as within-individual associations rather than definitive causal effects.

### Study size

The study size was determined by the number of women aged 15 years and older in HILDA waves 9–22 with valid information on disaster exposure. Among 15,600 women aged 15 years and older observed in survey waves 9–22, 1,775 (11.38%) had no valid disaster-exposure response in any eligible survey wave and were excluded. This left 13,825 women with at least one valid disaster-exposure response for the principal descriptive sample. The main descriptive sample contained 111,251 person-wave observations contributed by 13,825 women. Of these observations, 1,834 (1.65%) represented survey waves in which disaster exposure was reported.

Of the 13,825 women with at least one valid disaster-exposure response, 12,343 never reported exposure, 1,216 reported exposure in one survey wave, and 266 reported exposure in two or more survey waves. Thus, 1,482 women reported disaster exposure at least once during follow-up.

Outcome-specific availability and item non-response resulted in analytical samples of 110,851 observations from 13,805 women for the MHI-5, 110,183 observations from 13,796 women for the SF-36 General Health, and 55,252 observations from 12,797 women for the K10. The K10 analytical sample was smaller because this measure was available in fewer survey waves than the MHI-5 and SF-36 General Health measures.

### Statistical analysis

Within-individual associations between disaster exposure and each health outcome were estimated using individual fixed-effects panel regression models with survey-wave fixed effects. Robust standard errors were clustered at the individual level. Separate models were estimated for mental health, general health, and psychological distress.

The baseline model included only disaster exposure, individual fixed effects, and survey wave fixed effects:

$$y_{\mathrm{it}}=\beta_{1}\mathrm{Disaste}r_{\mathrm{it}}+\lambda_{t}+\alpha_{i}+\varepsilon_{\mathrm{it}}$$

where $y_{\mathrm{it}}$ represents the health outcome for woman iat survey wave *t*; $\beta_{1}\mathrm{Disaste}r_{\mathrm{it}}$ is the time-varying disaster-exposure indicator; $\lambda_{t}$ represents survey wave fixed effects; $\alpha_{i}$ represents individual fixed effects; and $\varepsilon_{\mathrm{it}}$ is the error term.

A secondary specification additionally included time-varying long-term illness:

$$y_{\mathrm{it}}=\beta_{1}\mathrm{Disaste}r_{\mathrm{it}}+\mathrm{\gamma LongIllnes}s_{\mathrm{it}}+\lambda_{t}+\alpha_{i}+\varepsilon_{\mathrm{it}}$$

In both models, $\beta_{1}$ represents the estimated within-individual difference in the outcome between survey waves in which a woman reported disaster exposure and survey waves in which she reported no exposure, after accounting for common survey-wave effects. In the secondary specification, the estimate additionally accounts for time-varying long-term illness.

As a first robustness analysis, the fixed-effects models were repeated in a sample restricted to women who reported disaster exposure in no more than one survey wave. This analysis examined whether the main findings were sensitive to the inclusion of women with repeated reported exposures.

Dynamic associations surrounding reported disaster exposure were examined using the multiple-period difference-in-differences estimator developed by Callaway and Sant’Anna, implemented using the outcome-regression method and without additional time-varying covariates [27]. Consistent with the single-exposure sensitivity analysis, these models were restricted to women reporting disaster exposure in no more than one survey wave. Women were assigned to treatment cohorts according to the survey wave in which they reported exposure, while women who never reported exposure served as the comparison group. The group-time average treatment effect was defined as:

$$\mathrm{ATT}\left(g,t\right)=E\left[\mathrm{Yt}\left(1\right)-\mathrm{Yt}\left(0\right)|G=g\right])$$

where $\mathrm{ATT}\left(g,t\right)\;$ represents the average difference between the observed outcome under exposure and the counterfactual outcome without exposure in survey wave t for women whose first reported exposure occurred in survey wave g.

Women whose first reported exposure occurred before they contributed an untreated observation were not used in comparisons requiring a pre-exposure period. The estimator accommodated the unbalanced panel using observations available for the relevant group-time comparisons.

The group-time estimates were aggregated according to event time, defined as:

e=t−g

where e=t−g represents the number of survey periods before or after the first reported disaster exposure. Dynamic estimates were presented for four periods before exposure through four periods after exposure $\left(e=-4\mathrm{toe}=4\right)$. The endpoints were not binned; estimates outside the displayed event-time window were omitted rather than combined with the estimates at e=−4ore=4. In addition to event-time-specific estimates, aggregated average pre-treatment and exposure/post-treatment estimates were reported for each outcome. Joint pre-trend tests assessed the null hypothesis that all available pre-exposure group-time coefficients were equal to zero; these tests therefore used the full set of available pre-treatment comparisons rather than only the four event-time estimates displayed in the figure. The event-study estimates were interpreted primarily as robustness evidence concerning pre-exposure patterns and the timing of associations rather than as evidence of persistent causal effects.

Finally, the fixed-effects and event-study analyses were repeated for women aged 18 years and older. This sensitivity analysis assessed whether the findings depended on including women aged 15–17 years in the principal analytical sample.

To examine potential differential attrition, wave-to-wave retention was compared between exposed and unexposed observations. Logistic regression models assessed whether reported disaster exposure was associated with participation in the subsequent survey wave, adjusting for survey-wave effects and clustering standard errors at the individual level. An interaction between disaster exposure and survey wave was additionally tested to assess whether differential retention varied over time.

The panel was unbalanced, and sample sizes differed across outcomes because of item non-response and differences in the survey waves in which particular outcomes were collected. All estimated coefficients were interpreted as associational rather than causal. All analyses were conducted using Stata version 15 (StataCorp, College Station, TX, USA).

## Results

### Descriptive characteristics

Table 1 presents descriptive characteristics for women aged 15 years and older across survey waves 9–22. The sample contained 111,251 person-wave observations with valid disaster-exposure information, of which 1,834 (1.65%) were classified as disaster-exposed and 109,417 (98.35%) as non-exposed. Outcome-specific sample sizes varied because of item non-response and because the K10 Psychological Distress Scale was administered only in selected survey waves.

**Table 1.** Descriptive characteristics of women aged 15 years and older, by disaster-exposure status.

Characteristic	Non-exposed	Exposed	Overall
Mental health (MHI-5), mean (SD)	71.76 (18.13)	66.88 (20.02)	71.68 (18.17)
Observations	109,027	1,824	110,851
Psychological distress (K10), mean (SD)	16.80 (7.11)	18.88 (8.77)	16.83 (7.14)
Observations	54,359	893	55,252
General health (SF-36 GH), mean (SD)	66.94 (21.33)	62.28 (22.67)	66.86 (21.36)
Observations	108,373	1,810	110,183
Age, years, mean (SD)	45.82 (19.02)	45.19 (18.26)	45.81 (19.01)
Observations	109,417	1,834	111,251
Long-term illness, %	24.13	30.48	24.24
Observations	109,246	1,831	111,077
Disaster-exposure observations, n (%)	109,417 (98.35%)	1,834 (1.65%)	111,251 (100%)
Unique women	12,343	1,482	13,825

Notes: Statistics are unadjusted. The sample includes women aged 15 years and older in survey waves 9–22 with valid disaster-exposure information. MHI-5 and SF-36 GH scores range from 0 to 100, with higher scores indicating better health. K10 scores range from 10 to 50, with higher scores indicating greater psychological distress. Sample sizes differ because of outcome-specific missingness and because K10 was administered only in selected survey waves. The exposed and non-exposed individual categories indicate whether a woman reported exposure at least once during follow-up.

The mean SF-36 Mental Health Index (MHI-5) score was 71.68 (SD = 18.17; *N* = 110,851), while the mean SF-36 General Health score was 66.86 (SD = 21.36; *N* = 110,183). The mean K10 psychological distress score was 16.83 (SD = 7.14; *N* = 55,252). The mean age was 45.81 years (SD = 19.01), and 24.24% of observations indicated the presence of a long-term illness or health condition.

Unadjusted differences were evident by disaster-exposure status. During disaster-exposed periods, women reported lower mean MHI-5 scores than during non-exposed periods (66.88 versus 71.76), lower mean general-health scores (62.28 versus 66.94), and higher mean K10 scores (18.88 versus 16.80). Long-term illness was also more prevalent among disaster-exposed observations than among non-exposed observations (30.48% versus 24.13%). Mean age was similar across exposure states (45.19 years among exposed observations and 45.82 years among non-exposed observations).

These descriptive differences are unadjusted and should not be interpreted as causal effects. The subsequent fixed-effects models use within-individual variation in disaster exposure over time while accounting for time-invariant individual characteristics and common survey-wave effects.

### Disaster exposure and women’s health

Tables 2–4 report individual fixed-effects estimates of the association between disaster exposure and the three health outcomes. All models include individual and survey wave fixed effects, with standard errors clustered at the individual level. The baseline models estimate the within-individual association between disaster exposure and health. The second specification additionally includes contemporaneous long-term illness as a sensitivity analysis.

**Table 2.** Fixed-effects estimates of the association between disaster exposure and women’s mental health (MHI-5).

Variable	(1) Baseline FE	(2) + Long-term illness (sensitivity)
Weather-related disaster exposure	−0.859*	−0.849*
	(0.334)	(0.333)
95% CI	[−1.513, −0.204]	[−1.503, −0.196]
Long-term illness	—	−2.285***
		(0.151)
Individual fixed effects	Yes	Yes
Survey-wave fixed effects	Yes	Yes
Observations	110,851	110,678
Individuals	13,805	13,789
Within R^2^	0.010	0.014

Notes: Cluster-robust standard errors at the individual level are reported in parentheses. The sample includes women aged 15 years and older with valid disaster-exposure and outcome information. Model 2 additionally includes contemporaneous long-term illness and is treated as a sensitivity specification. Higher MHI-5 scores indicate better mental health. †*p* < 0.10; *p < 0.05; **p < 0.01; ***p < 0.001.

**Table 3.** Fixed-effects estimates of the association between disaster exposure and women’s psychological distress (K10).

Variable	(1) Baseline FE	(2) Long-term illness (sensitivity)
Weather-related disaster exposure	0.326†	0.323†
	(0.188)	(0.188)
95% CI	[−0.043, 0.695]	[−0.046, 0.692]
Long-term illness	—	1.019***
		(0.078)
Individual fixed effects	Yes	Yes
Survey-wave fixed effects	Yes	Yes
Observations	55,252	55,153
Individuals	12,797	12,783
Within R^2^	0.018	0.023

Notes: Cluster-robust standard errors at the individual level are reported in parentheses. The sample includes women aged 15 years and older with valid disaster-exposure and outcome information. Model 2 additionally includes contemporaneous long-term illness and is treated as a sensitivity specification. Higher K10 scores indicate greater psychological distress. K10 was administered only in selected survey waves. †*p* < 0.10; *p < 0.05; **p < 0.01; ***p < 0.001.

**Table 4.** Fixed-effects estimates of the association between disaster exposure and women’s general health (SF-36 GH).

Variable	(1) Baseline FE	(2) Long-term illness (sensitivity)
Weather-related disaster exposure	−0.830**	−0.828**
	(0.313)	(0.310)
95% CI	[−1.443, −0.217]	[−1.437, −0.220]
Long-term illness	—	−5.568***
		(0.166)
Individual fixed effects	Yes	Yes
Survey-wave fixed effects	Yes	Yes
Observations	110,183	110,009
Individuals	13,796	13,780
Within R^2^	0.030	0.050

Notes: Cluster-robust standard errors at the individual level are reported in parentheses. The sample includes women aged 15 years and older with valid disaster-exposure and outcome information. Model 2 additionally includes contemporaneous long-term illness and is treated as a sensitivity specification. Higher SF-36 GH scores indicate better self-reported general health. †*p* < 0.10; *p < 0.05; **p < 0.01; ***p < 0.001.

### Mental health

Table 2 presents the results for mental health measured using the MHI-5. In the baseline fixed-effects model, disaster exposure was associated with a 0.859-point reduction in MHI-5 scores (β = −0.859, SE = 0.334, 95% CI: −1.513 to −0.204, *p* = 0.010). The model included 110,851 person-wave observations from 13,805 women.

The estimated association was essentially unchanged after including long-term illness (β = −0.849, SE = 0.333, 95% CI: −1.503 to −0.196, *p* = 0.011). Long-term illness was associated with lower MHI-5 scores (β = −2.285, SE = 0.151, *p* < 0.001), while its inclusion did not materially alter the estimated association between disaster exposure and mental health.

### Psychological distress

Table 3 presents the results for psychological distress measured using the K10. In the baseline fixed-effects model, disaster exposure was associated with a 0.326-point increase in psychological distress (β = 0.326, SE = 0.188, 95% CI: −0.043 to 0.695, *p* = 0.084). The model included 55,252 person-wave observations from 12,797 women. Although the coefficient was in the adverse direction, the confidence interval included zero and the association did not reach the conventional 5% threshold for statistical significance.

Including long-term illness produced little change in the estimated disaster coefficient (β = 0.323, SE = 0.188, 95% CI: −0.046 to 0.692, *p* = 0.086). Long-term illness was associated with higher K10 scores (β = 1.019, SE = 0.078, *p* < 0.001). Thus, the K10 estimates were consistent with higher psychological distress during disaster-exposed periods, but the association was not statistically significant at the 5% level.

### General health

Table 4 presents the results for general health measured using the SF-36 General Health scale. In the baseline fixed-effects model, disaster exposure was associated with a 0.830-point reduction in general-health scores (β = −0.830, SE = 0.313, 95% CI: −1.443 to −0.217, *p* = 0.008). The model included 110,183 person-wave observations from 13,796 women.

The estimated association remained virtually unchanged after including long-term illness (β = −0.828, SE = 0.310, 95% CI: −1.437 to −0.220, *p* = 0.008). Long-term illness was independently associated with lower general-health scores (β = −5.568, SE = 0.166, *p* < 0.001). The similarity of the disaster-exposure estimates across specifications indicates that adjustment for contemporaneous long-term illness did not materially alter the estimated association with general health.

### Sensitivity to repeated exposure

Appendix Table A1 presents sensitivity analyses restricted to women reporting disaster exposure in no more than one survey wave. Among women with at least one valid exposure response, 12,343 reported no exposure, 1,216 reported exposures in one survey wave, and 266 reported exposures in two or more survey waves.

The adverse association with the MHI-5 remained statistically significant and was somewhat larger than in the primary analysis (β = −1.008, SE = 0.392, 95% CI: −1.776 to −0.239, *p* = 0.010). The general-health association also remained negative but was smaller in magnitude and less precisely estimated (β = −0.737, SE = 0.368, 95% CI: −1.459 to −0.015, *p* = 0.046). In contrast, the K10 coefficient was attenuated and statistically nonsignificant (β = 0.180, SE = 0.214, 95% CI: −0.239 to 0.600, *p* = 0.400). These findings indicate that the MHI-5 association was robust to excluding women with repeated exposure, while the general-health association was smaller and less precisely estimated. The K10 association remained statistically nonsignificant.

### Dynamic event-study estimates

Figure 1 presents dynamic estimates from the Callaway–Sant’Anna difference-in-differences estimator, with summary estimates reported in Appendix Table A2. These analyses used women who never reported disaster exposure during the analytical period as the comparison group, restricted the sample to women reporting exposure in no more than one survey wave, and excluded person-wave observations with missing disaster-exposure or outcome information. Estimates are presented for four event-time periods before exposure, the exposure period, and four periods after exposure.

**Figure 1.**
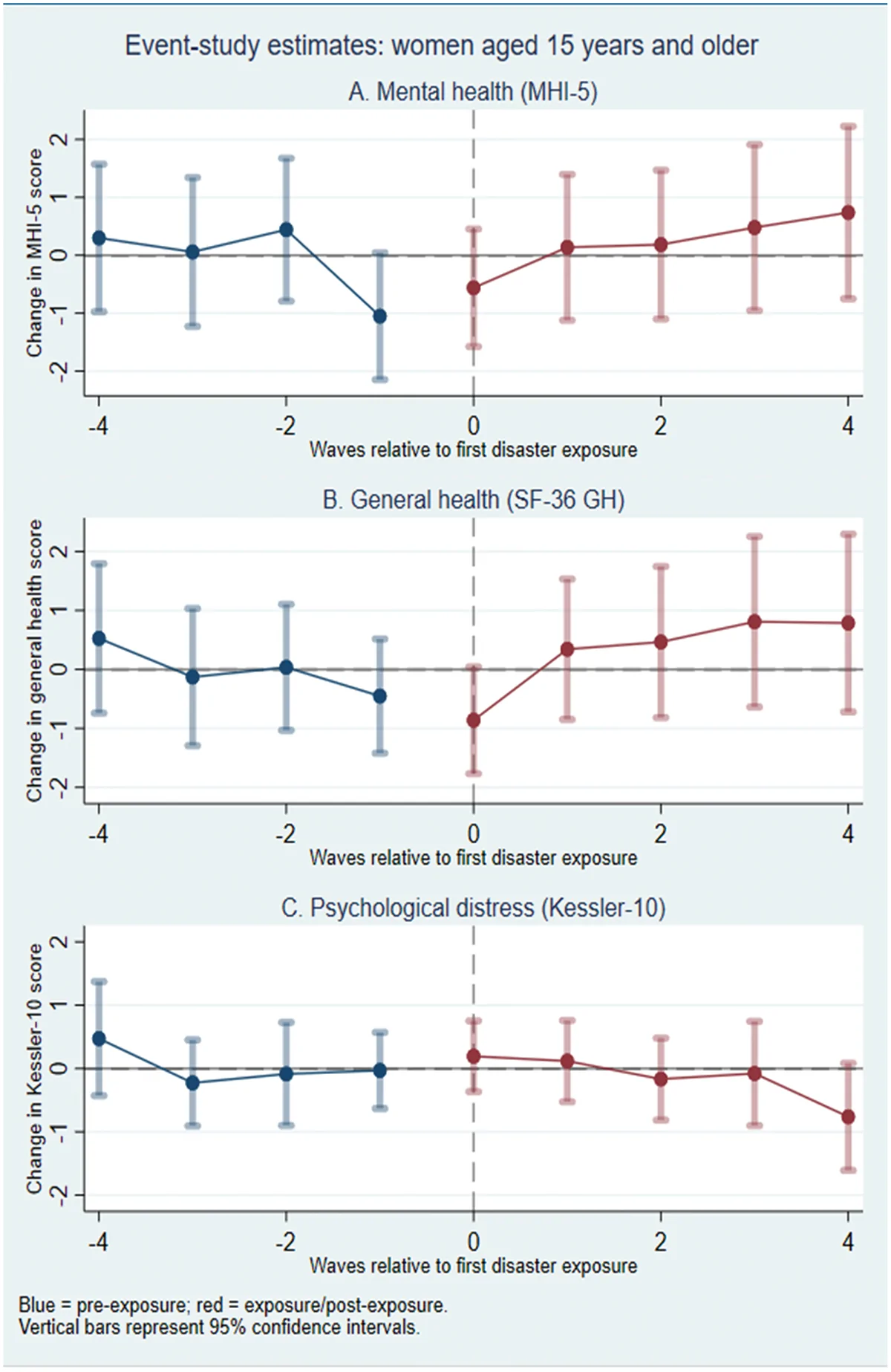
Event-study estimates of the association between disaster exposure and women’s health outcomes. Notes: This figure presents dynamic estimates from the Callaway–Sant’Anna multiple-period difference-in-differences estimator for mental health (MHI-5), general health (SF-36 General Health), and psychological distress (K10). Women were assigned to treatment cohorts according to the survey wave of their first reported disaster exposure, while women who never reported exposure during the analytical period served as the comparison group. The analysis was restricted to women reporting disaster exposure in no more than one survey wave. Event time is measured relative to the first reported exposure, with the immediately preceding period (*e* = –1) serving as the reference period and *e* = 0 representing the exposure wave. The endpoints were not binned; estimates outside the displayed window were omitted. Vertical bars represent 95% confidence intervals, the vertical dashed line identifies the exposure wave, and the horizontal line denotes zero. Because the estimator aggregates cohort-specific group-time comparisons in an unbalanced panel, the number and composition of women contributing to the estimates vary across event times. Total outcome-specific analytical sample sizes are reported in Appendix Table A2.

For MHI-5, the average pre-treatment estimate was −0.062 (p=0.757), while the average exposure/post-treatment estimate was 0.196 (p=0.685). The estimate in the exposure period was negative but statistically nonsignificant (β=−0.560, SE = 0.518, 95% CI: −1.576 to 0.455, p=0.279). None of the subsequent post-exposure estimates was statistically significant.

For general health, the average pre-treatment estimate was −0.005 (p=0.978), while the average exposure/post-treatment estimate was 0.308 (p=0.529). The exposure-period estimate suggested lower general health but did not reach the conventional 5% significance threshold (β=−0.862, SE = 0.462, 95% CI: −1.767 to 0.043, p=0.062). Subsequent post-exposure estimates were statistically nonsignificant and did not indicate persistent deterioration in general health.

For K10, the average pre-treatment estimate was 0.032 (p=0.835), while the average exposure/post-treatment estimate was −0.139 (p=0.493). The exposure-period estimate was small and statistically nonsignificant (β=0.193, SE = 0.285, 95% CI: −0.366 to 0.752, p=0.498). None of the estimates from the exposure period through four post-exposure periods reached statistical significance at the 5% level. The estimate at four post-exposure periods was weakly significant at the 10% level (β=−0.762, SE = 0.431, 95% CI: −1.608 to 0.083, p=0.077) but was in the direction of lower rather than higher psychological distress.

Overall, the event-study estimates provided no consistent evidence of persistent post-exposure changes in the three health outcomes. However, the pre-trend findings, particularly for MHI-5, limit causal interpretation of the dynamic estimates.

### Assessment of pre-treatment trends

Formal joint tests produced different results across the three outcomes (Appendix Table A2). For MHI-5, the global test rejected the null hypothesis that all available pre-exposure group-time coefficients were jointly equal to zero at the 5% level (χ^2^(78) = 100.40, *p* = 0.045). This indicates evidence of differential pre-exposure patterns and limits causal interpretation of the MHI-5 event-study estimates.

For general health, the global test did not reject the null hypothesis at the 5% level, although the result was close to this threshold (χ^2^(78) = 98.34, *p* = 0.060). The general-health event-study estimates should therefore also be interpreted cautiously. For K10, the global test was statistically non-significant (χ^2^(36) = 42.33, *p* = 0.217), providing less evidence of differential pre-exposure patterns.

These tests used all available pre-exposure group-time comparisons rather than only the four pre-exposure estimates displayed in Figure 1. Overall, the pre-trend results reinforce the interpretation of the event-study analyses as descriptive robustness evidence concerning timing rather than as estimates of causal effects.

### Sensitivity to the minimum-age restriction

Appendix Table A3 presents fixed-effects sensitivity analyses restricted to women aged 18 years and older. The estimated MHI-5 association remained adverse and statistically significant in both the baseline model (β = −0.917, SE = 0.336, 95% CI: −1.576 to −0.258, *p* = 0.006) and the model including long-term illness (β = −0.908, SE = 0.335, 95% CI: −1.565 to −0.250, *p* = 0.007).

The general-health results were similarly robust. Disaster exposure was associated with a 0.815-point reduction in the baseline model (β = −0.815, SE = 0.319, 95% CI: −1.440 to −0.189, *p* = 0.011) and a 0.813-point reduction after including long-term illness (β = −0.813, SE = 0.316, 95% CI: −1.433 to −0.193, *p* = 0.010).

The age-18+ K10 coefficients remained positive but statistically nonsignificant in both the baseline model (β = 0.292, SE = 0.190, 95% CI: −0.081 to 0.665, *p* = 0.125) and the model including long-term illness (β = 0.290, SE = 0.190, 95% CI: −0.083 to 0.663, *p* = 0.128). Overall, the similarity between the age-15+ and age-18+ estimates indicates that the principal fixed-effects findings were not sensitive to the minimum-age threshold.

The age-18+ event-study estimates are presented in Appendix Figure A1. Average post-treatment estimates were statistically nonsignificant for MHI-5 (0.091, *p* = 0.853), general health (0.309, *p* = 0.529), and K10 (−0.103, *p* = 0.613). The global pre-trend test was not statistically significant for MHI-5 (χ^2^(78) = 96.96, *p* = 0.072) or K10 (χ^2^(36) = 44.51, *p* = 0.156). However, the global test for general health rejected the null hypothesis at the 5% level (χ^2^(78) = 101.75, *p* = 0.037), indicating that the age-18+ general-health event-study estimates should be interpreted cautiously.

### Supplementary adjustment for geographic and socioeconomic characteristics

Appendix Tables A4–A6 present supplementary fixed-effects models incorporating geographic remoteness, educational attainment, and labour-force status. All models retained individual and survey-wave fixed effects and adjusted for contemporaneous long-term illness. These characteristics were examined separately because they may change over time and could also represent pathways through which disaster exposure influences health.

Adding geographic remoteness produced little change in the disaster-exposure coefficients (Appendix Table A4). Disaster exposure remained associated with lower MHI-5 scores (β = −0.867, SE = 0.333, *p* = 0.009) and lower general-health scores (β = −0.844, SE = 0.310, *p* = 0.007). The K10 coefficient remained positive but did not reach the 5% significance threshold (β = 0.333, SE = 0.188, *p* = 0.076). Compared with periods of residence in major cities, residence in inner-regional areas was associated with higher MHI-5 scores and lower K10 scores. Residence in outer-regional, remote or very remote areas was associated with higher MHI-5 and general-health scores and lower K10 scores. These coefficients represent within-woman changes associated with changes in remoteness category rather than cross-sectional differences between urban and regional populations.

Including educational attainment also did not materially alter the disaster-exposure estimates (Appendix Table A5). The disaster coefficient was −0.859 for MHI-5 (SE = 0.333, *p* = 0.010), 0.325 for K10 (SE = 0.188, *p* = 0.084), and −0.815 for general health (SE = 0.310, *p* = 0.009). Because educational attainment changed for relatively few women during the panel, the education coefficients were identified only by within-woman changes in recorded attainment and should not be interpreted as conventional differences between education groups.

The results were similarly stable after including labour-force status (Appendix Table A6). Disaster exposure remained associated with lower MHI-5 scores (β = −0.856, SE = 0.334, *p* = 0.010) and lower general-health scores (β = −0.828, SE = 0.310, *p* = 0.008), while its association with K10 scores remained positive but statistically nonsignificant at the 5% level (β = 0.328, SE = 0.188, *p* = 0.081). Relative to periods of employment, periods of unemployment were associated with lower MHI-5 scores and higher K10 scores. Periods outside the labour force were associated with lower general-health scores but were not significantly associated with MHI-5 or K10 scores.

Overall, the estimated disaster-exposure coefficients were highly consistent across the remoteness, education, and labour-force specifications. The estimated disaster-exposure coefficients were largely unchanged across these supplementary specifications.

### Wave-by-wave attrition analysis

Appendix Table A7 presents the wave-by-wave attrition diagnostic. Among 103,352 eligible person-wave observations from waves 9–21, 98,042 (94.86%) were observed in the immediately following survey wave, while 5,310 (5.14%) were not observed. Across individual wave transitions, retention ranged from 93.22% to 95.84% among non-exposed observations and from 91.53% to 98.10% among disaster-exposed observations.

After adjustment for survey wave, the estimated probability of next-wave retention was 94.87% among non-exposed observations and 94.62% among exposed observations. The adjusted difference was −0.24% points (95% CI: −1.41 to 0.92, *p* = 0.683), providing no evidence that disaster exposure was associated with subsequent attrition overall.

Twelve of the 13 wave-specific comparisons were statistically nonsignificant. For the wave 19–20 transition, disaster-exposed observations had a 3.78-percentage-point higher probability of retention (*p* = 0.005). However, a joint test indicated that exposure-related retention differences did not vary significantly across survey waves (χ^2^(12) = 8.66, *p* = 0.732). Taken together, the results provided no evidence of systematic differential attrition according to disaster-exposure status.

### Summary of findings

Overall, disaster exposure was associated with modest reductions in women’s mental and general health in the primary fixed-effects analyses. The estimated increase in psychological distress measured by the K10 did not reach statistical significance at the 5% level. The MHI-5 and general-health associations remained similar after adjusting separately for geographic remoteness, educational attainment, and labour-force status, as well as after restricting the analysis to women aged 18 years and older. The MHI-5 association also remained statistically significant after excluding women who reported exposure in more than one survey wave. The corresponding general-health estimate remained negative but was smaller and less precisely estimated, while the K10 association remained statistically nonsignificant.

The event-study analyses provided little evidence of persistent post-exposure changes. The pre-treatment tests indicated that the dynamic MHI-5 and general-health estimates should be interpreted cautiously. Finally, the wave-by-wave attrition analysis found no evidence that disaster exposure systematically predicted participation in the following survey wave. Collectively, these findings support modest contemporaneous within-individual associations between reported disaster exposure and poorer mental and general health, but do not establish persistent or causal health effects.

## Discussion

This study provides longitudinal evidence on the association between reported disaster exposure and women’s health using data from the Household, Income and Labour Dynamics in Australia Survey and individual fixed-effects models. By exploiting within-person variation over time and controlling for common temporal shocks through survey wave fixed effects, the analysis accounts for time-invariant individual characteristics and factors common to respondents within each survey wave. Reported disaster exposure was associated with modest declines in mental and general health. Psychological distress was also higher during exposed periods, although this association did not reach conventional statistical significance in the primary specification. These findings are broadly consistent with recent Australian longitudinal evidence showing that disaster exposure may be associated with poorer mental health, while also highlighting the importance of accounting for repeated exposure and heterogeneity in disaster experiences [13].

The magnitude of the estimated associations was small relative to the ranges and observed variation of the health measures. This distinction between statistical evidence and substantive magnitude is important, particularly given the large analytical sample. The findings therefore should not be interpreted as demonstrating large individual-level changes in health following disaster exposure. Nevertheless, relatively small average changes may remain relevant at a population level when exposure affects substantial numbers of people, although the present study did not directly assess clinical significance or population-level health burden.

The sensitivity analyses generally supported the direction of the primary findings while also highlighting uncertainty in their magnitude and statistical precision. Including time-varying long-term illness produced little change in the estimated associations across the three health outcomes. Because long-term illness may itself be influenced by disaster exposure, however, the illness-adjusted specification was treated as a sensitivity analysis rather than the primary model. Restricting the sample to women reporting disaster exposure in no more than one survey wave similarly produced evidence of poorer mental and general health, whereas the estimate for psychological distress remained statistically uncertain. Analyses restricted to women aged 18 years and older produced broadly comparable fixed-effects estimates. The estimated disaster-exposure associations were also largely unchanged in supplementary specifications that included geographic remoteness, educational attainment, and labour-force status.

The multiple-period difference-in-differences event-study analysis provided a complementary assessment of health patterns surrounding reported disaster exposure. Across the three outcomes, the aggregated average pre-exposure estimates were small and statistically nonsignificant. However, the formal joint test rejected the null hypothesis that all available pre-exposure group-time coefficients were equal to zero for MHI-5, indicating evidence of differential pre-exposure patterns. The corresponding test for general health was close to the 5% significance threshold, while the K10 test was statistically nonsignificant. These findings limit causal interpretation of the MHI-5 and general-health dynamic estimates and demonstrate that nonsignificant individual pre-exposure estimates do not establish the absence of differential pre-exposure trends. The post-exposure estimates did not provide consistent evidence of persistent deterioration in health over subsequent survey waves. In the age-18 sensitivity analysis, the joint pre-trend test for general health was also statistically significant, further limiting causal interpretation of the corresponding dynamic estimates. Accordingly, the event-study findings are interpreted as descriptive evidence concerning timing and pre-exposure patterns rather than as establishing sustained or causal health effects. Recent longitudinal research has similarly demonstrated that post-disaster psychological adjustment may follow heterogeneous trajectories over time, reinforcing the value of examining dynamic patterns rather than assuming uniform or persistent responses following exposure [6].

These findings add longitudinal evidence to research examining disaster exposure and women’s health. Several pathways may plausibly contribute to health changes surrounding disaster exposure, including psychological stress, housing or financial disruption, changes in employment and caregiving demands, reduced social support, and interruptions to healthcare or other services. Other unmeasured pathways, including sleep disruption and post-traumatic stress reactions, may also contribute to health changes following disaster exposure. Recent research has similarly emphasised the multiple social and psychological pathways through which disasters and climate-related events may affect mental health and wellbeing [5,20]. These mechanisms were not directly measured in the present analysis and therefore should be regarded as potential explanations rather than established pathways. Moreover, vulnerability is unlikely to be uniform among women and may vary according to socioeconomic circumstances, age, health status, family responsibilities, housing security, location, and access to recovery resources. This interpretation is consistent with recent systematic-review evidence demonstrating that disaster-related mental health outcomes may differ substantially across socially, economically, geographically, and medically vulnerable populations [22].

From an applied health economics perspective, even modest changes in mental and general health may have potential implications for healthcare utilisation, productivity, labour supply, household wellbeing, and health-system demand. However, these downstream outcomes were not directly examined in this study. The findings therefore identify health changes that may be economically relevant rather than demonstrating economic costs attributable to disaster exposure. Future research linking disaster-related health changes to healthcare utilisation, employment, earnings, productivity, and quality of life would be valuable for estimating the broader economic burden associated with disasters.

The findings also have potential implications for disaster preparedness and recovery planning. The observed associations with mental and general health support consideration of health and psychological wellbeing within disaster-response frameworks. However, the study did not evaluate specific healthcare services, recovery programmes, or policy interventions and therefore cannot determine which particular models of support are most effective. Policy implications should accordingly be interpreted as informing the need for further attention to health within disaster preparedness and recovery rather than identifying specific interventions.

The focus on women reflects evidence that social and economic circumstances, including caregiving responsibilities, labour-market conditions, household resources, and access to recovery support, may shape women’s experiences during and after disasters. Recent systematic-review evidence has similarly highlighted the importance of gendered social roles and inequalities in shaping women’s experiences during disaster emergencies [20]. However, because the analysis did not directly compare women with men, the findings should not be interpreted as demonstrating that women experience greater disaster-related health changes than men. Rather, they provide evidence concerning within-person health changes in a female-specific sample. Future comparative research could examine whether the magnitude or pattern of these associations differs by gender and across groups of women with different socioeconomic and demographic characteristics.

Several limitations should be acknowledged. First, disaster exposure was self-reported and measured at the survey-wave level, creating potential for recall or reporting error and uncertainty regarding the precise timing of exposure relative to the health assessments. Because exposure and health information were collected within the same survey framework, the temporal ordering of changes cannot always be determined precisely, and reporting processes may contribute to observed associations. Second, the binary exposure measure does not distinguish disaster type, intensity, duration, displacement, injury, financial loss, or other dimensions of disaster severity. Such measurement limitations may result in exposure misclassification and obscure heterogeneous health responses. If exposure misclassification is predominantly nondifferential with respect to health outcomes, it would generally be expected to attenuate associations towards zero. However, because exposure was self-reported and reporting may be related to health status, the direction of any resulting bias cannot be determined with certainty. Recent Australian longitudinal research has also highlighted the importance of distinguishing repeated disaster exposure when examining mental health outcomes [13].

Third, some women reported disaster exposure in more than one survey wave. Although a single-exposure sensitivity analysis was undertaken, the primary fixed-effects estimates represent average within-person associations across exposed and unexposed observations and cannot distinguish the consequences of a single from repeated disaster experiences. Fourth, although individual fixed effects account for time-invariant observed and unobserved characteristics, residual time-varying confounding cannot be excluded. Changes in household circumstances, income, employment, relocation, healthcare access, family circumstances, or concurrent local events may coincide with both reported disaster exposure and health. The relatively low within-person R^2^ values indicate that the models explain only a small proportion of year-to-year variation in health. Although this is not uncommon in individual fixed-effects analyses of health outcomes, it highlights the importance of other unmeasured time-varying determinants of health and further supports cautious interpretation of the estimated disaster-exposure associations.

Fifth, the panel was unbalanced and participants contributed different numbers of observations over time. Although the wave-by-wave analysis found no evidence of systematic differential attrition according to disaster-exposure status, attrition and item non-response may still affect the analytical samples if continued participation or missingness is related to unobserved health or disaster-related circumstances. Outcome availability also differed across survey waves, particularly for the Kessler Psychological Distress Scale, resulting in a smaller analytical sample for this outcome. Sixth, longitudinal survey weights were not applied to the fixed-effects analyses; consequently, although the Household, Income and Labour Dynamics in Australia Survey was designed as a nationally representative household panel, the analytical samples and particularly the subset contributing within-person exposure variation should not be interpreted as necessarily representative of all Australian women.

Finally, the event-study estimates were imprecise for several event times. The statistically significant joint pre-trend test for MHI-5 in the principal sample indicated differential pre-exposure patterns, while the general-health test was close to the 5% significance threshold. The age-18 sensitivity analysis also produced a statistically significant general-health pre-trend test. These findings limit causal interpretation of the corresponding event-study estimates. More generally, nonsignificant pre-exposure estimates do not establish the absence of differential pre-exposure trends. The results should therefore be interpreted as associational rather than causal. Generalisability may also be limited beyond the Australian institutional and disaster-response context.

Despite these limitations, this study contributes longitudinal evidence on within-person changes in women’s mental and general health surrounding reported disaster exposure. The combination of individual fixed-effects models, long-term illness and single-exposure sensitivity analyses, multiple-period difference-in-differences event studies, formal pre-trend testing, and age-restricted sensitivity analyses provides complementary evidence while allowing the remaining uncertainty and limitations of the observational design to be made explicit.

## Conclusion

Reported disaster exposure was associated with modest declines in women’s mental and general health in longitudinal fixed-effects analyses. Psychological distress was higher during exposed periods, although the association was statistically uncertain in the primary specification. Sensitivity analyses generally supported the direction of the mental and general health findings, while the event-study analysis did not provide consistent evidence that adverse health changes persisted over subsequent survey waves.

These findings suggest that women’s mental and general health should be considered alongside physical and material consequences when assessing the broader implications of disasters. Potential downstream consequences for healthcare utilisation, productivity, labour supply, and economic welfare warrant further investigation but were not directly assessed in this study. Future research should examine heterogeneity according to disaster characteristics and population circumstances, compare patterns across gender groups, and investigate healthcare utilisation, labour-market outcomes, and other potential economic consequences.

Overall, the study provides evidence of modest within-person health changes associated with reported disaster exposure among women while highlighting the importance of cautious interpretation given uncertainty regarding exposure timing, residual time-varying confounding, attrition, and heterogeneity in disaster experiences. These findings support continued consideration of mental and general health within disaster preparedness and recovery research and policy.

## Supplementary Material

Disaster exposure_Strobe_GHA.docx

Supplementary Material_Tables_GHA.docx

## Data Availability

The data used in this study are from the Household, Income and Labour Dynamics in Australia (HILDA) Survey. HILDA unit-record data are not publicly available for unrestricted download. Access is available to approved researchers through the Australian Data Archive (ADA) Dataverse, subject to application, approval, and compliance with the data access and use requirements of the Australian Government Department of Social Services. Information on HILDA data and access procedures is available through the Department of Social Services HILDA webpage and the Australian Data Archive Dataverse.
Department of Social Services – HILDA Survey and data access Australian Data Archive Dataverse
