# Supplementary material for: Disaster exposure and women’s mental and general health: evidence from longitudinal data
Source: Glob Health Action. 2026 Oct 27;19(1):2752483. doi: 10.1080/16549716.2026.2752483 (PMC13715346; doi:10.1080/16549716.2026.2752483)
Supplement: Supplementary file 1 [file ZGHA_A_2752483_SM6029.docx]

**Supplementary Material**

*for*

# Disaster Exposure and Women’s Mental and General Health: Evidence from Longitudinal Data

| **Appendix Table A1. Sensitivity analysis restricted to women reporting disaster exposure in no more than one survey wave** | | | | | | | |
| --- | --- | --- | --- | --- | --- | --- | --- |
| **Outcome** | **Weather-related disaster-exposure coefficient** | **SE** | **95% CI** | **p-value** | **Observations** | **Individuals** | **Within R²** |
| Mental health (MHI-5) | −1.008* | 0.392 | [−1.776, −0.239] | 0.010 | 107,830 | 13,539 | 0.010 |
| Psychological distress (K10) | 0.180 | 0.214 | [−0.239, 0.600] | 0.400 | 53,745 | 12,531 | 0.018 |
| General health (SF-36 GH) | −0.737* | 0.368 | [−1.459, −0.015] | 0.046 | 107,179 | 13,530 | 0.030 |
| *Notes:* Estimates are from individual fixed-effects models that include survey-wave fixed effects. Standard errors are clustered at the individual level. The sample includes women aged 15 years and older and is restricted to women reporting disaster exposure in no more than one survey wave. These models correspond to the baseline fixed-effects specification and do not additionally adjust for long-term illness. Higher MHI-5 and SF-36 GH scores indicate better health, whereas higher K10 scores indicate greater psychological distress. † p < 0.10; * p < 0.05; ** p < 0.01; *** p < 0.001. | | | | | | | |

| **Appendix Table A2. Callaway–Sant’Anna event-study estimates and tests of pre-exposure trends** | | | | | | |
| --- | --- | --- | --- | --- | --- | --- |
| **Panel A. Summary estimates** | | | | | | |
| Outcome | Observations | Average pre-estimate | *p*-value | Average exposure/post-estimate | *p*-value | Global pre-trend test |
| Mental health (MHI-5) | 105,440 | −0.062 | 0.757 | 0.196 | 0.685 | χ²(78) = 100.40; *p* = 0.045 |
| General health (SF-36 GH) | 104,683 | −0.005 | 0.978 | 0.308 | 0.529 | χ²(78) = 98.34; *p* = 0.060 |
| Psychological distress (K10) | 50,916 | 0.032 | 0.835 | −0.139 | 0.493 | χ²(36) = 42.33; *p* = 0.217 |
| **Panel B. Event-time-specific estimates** | | | | | | |
| Event time | MHI-5 estimate (95% CI) | *p*-value | SF-36 GH estimate (95% CI) | *p*-value | K10 estimate (95% CI) | *p*-value |
| −4 | 0.300 [−0.973, 1.573] | 0.644 | 0.526 [−0.741, 1.793] | 0.416 | 0.473 [−0.427, 1.373] | 0.303 |
| −3 | 0.058 [−1.227, 1.343] | 0.929 | −0.129 [−1.292, 1.033] | 0.827 | −0.227 [−0.906, 0.452] | 0.512 |
| −2 | 0.443 [−0.790, 1.676] | 0.482 | 0.035 [−1.034, 1.105] | 0.949 | −0.086 [−0.899, 0.728] | 0.837 |
| −1 | −1.051 [−2.145, 0.044] | 0.060 | −0.452 [−1.420, 0.516] | 0.360 | −0.031 [−0.632, 0.570] | 0.920 |
| 0: exposure wave | −0.560 [−1.576, 0.455] | 0.279 | −0.862 [−1.767, 0.043] | 0.062 | 0.193 [−0.366, 0.752] | 0.498 |
| +1 | 0.138 [−1.121, 1.397] | 0.830 | 0.342 [−0.848, 1.532] | 0.573 | 0.117 [−0.525, 0.760] | 0.720 |
| +2 | 0.185 [−1.100, 1.469] | 0.778 | 0.465 [−0.817, 1.747] | 0.477 | −0.167 [−0.814, 0.480] | 0.613 |
| +3 | 0.480 [−0.953, 1.912] | 0.512 | 0.809 [−0.636, 2.254] | 0.273 | −0.078 [−0.902, 0.745] | 0.853 |
| +4 | 0.738 [−0.750, 2.226] | 0.331 | 0.787 [−0.720, 2.294] | 0.306 | −0.762 [−1.608, 0.083] | 0.077 |
| *Notes:* Estimates were obtained using the Callaway–Sant’Anna multiple-period difference-in-differences estimator with the outcome-regression method and without additional time-varying covariates. Women who never reported disaster exposure during the analytical period served as the comparison group. The sample includes women aged 15 years and older with valid disaster-exposure and outcome information and is restricted to women reporting disaster exposure in no more than one survey wave. Negative event times represent pre-exposure periods, event time zero represents the exposure wave, and positive event times represent post-exposure periods. The endpoints were not binned; estimates outside the displayed event-time window were omitted. The estimator accommodated the unbalanced panel using observations available for the relevant group-time comparisons. Higher MHI-5 and SF-36 GH scores indicate better health, whereas higher K10 scores indicate greater psychological distress. The global pre-trend test evaluates the null hypothesis that all available pre-exposure group-time estimates are jointly equal to zero. | | | | | | |

| **Appendix Table A3. Fixed-effects sensitivity analysis restricted to women aged 18 years and older** | | | | | | | |
| --- | --- | --- | --- | --- | --- | --- | --- |
| **Outcome** | **Model** | **Weather-related disaster-exposure coefficient (SE)** | **95% CI** | **p-value** | **Observations** | **Individuals** | **Within R²** |
| Mental health (MHI-5) | Baseline | −0.917** (0.336) | [−1.576, −0.258] | 0.006 | 106,000 | 13,181 | 0.010 |
| Mental health (MHI-5) | + Long-term illness | −0.908** (0.335) | [−1.565, −0.250] | 0.007 | 105,831 | 13,164 | 0.014 |
| Psychological distress (K10) | Baseline | 0.292 (0.190) | [−0.081, 0.665] | 0.125 | 52,829 | 12,247 | 0.017 |
| Psychological distress (K10) | + Long-term illness | 0.290 (0.190) | [−0.083, 0.663] | 0.128 | 52,731 | 12,231 | 0.023 |
| General health (SF-36 GH) | Baseline | −0.815* (0.319) | [−1.440, −0.189] | 0.011 | 105,332 | 13,171 | 0.030 |
| General health (SF-36 GH) | + Long-term illness | −0.813* (0.316) | [−1.433, −0.193] | 0.010 | 105,162 | 13,154 | 0.052 |
| *Notes:* All models include individual and survey-wave fixed effects. Standard errors are clustered at the individual level. Models labelled “+ Long-term illness” additionally include contemporaneous long-term illness and are treated as sensitivity specifications. Higher MHI-5 and SF-36 GH scores indicate better health, whereas higher K10 scores indicate greater psychological distress. † p < 0.10; * p < 0.05; ** p < 0.01; *** p < 0.001. | | | | | | | |

| **Appendix Table A4. Fixed-effects models additionally adjusting for geographic remoteness** | | | |
| --- | --- | --- | --- |
| **Variable** | **MHI-5** | **K10** | **General health** |
| Weather-related disaster exposure | −0.867** | 0.333† | −0.844** |
|  | (0.333) | (0.188) | (0.310) |
| Long-term illness | −2.282*** | 1.021*** | −5.568*** |
|  | (0.151) | (0.078) | (0.166) |
| Inner regional | 0.644* | −0.292* | 0.219 |
|  | (0.296) | (0.146) | (0.304) |
| Outer regional/remote/very remote | 1.162** | −0.780*** | 1.012* |
|  | (0.411) | (0.208) | (0.435) |
| Individual fixed effects | Yes | Yes | Yes |
| Survey-wave fixed effects | Yes | Yes | Yes |
| Observations | 110,648 | 55,143 | 109,979 |
| Individuals | 13,787 | 12,783 | 13,778 |
| Within R² | 0.014 | 0.024 | 0.050 |
| *Notes:* Cluster-robust standard errors at the individual level are reported in parentheses. All models include individual and survey-wave fixed effects and contemporaneous long-term illness. The reference category for geographic remoteness is major city. Remoteness coefficients represent within-woman associations arising from changes in residential remoteness over time and should not be interpreted as cross-sectional differences between geographic populations. Higher MHI-5 and SF-36 GH scores indicate better health, whereas higher K10 scores indicate greater psychological distress. † $p<0.10$; * $p<0.05$; ** $p<0.01$; *** $p<0.001$ | | | |

| **Appendix Table A5. Fixed-effects models additionally adjusting for educational attainment** | | | |
| --- | --- | --- | --- |
| **Variable** | **MHI-5** | **K10** | **General health** |
| Weather-related disaster exposure | −0.859** | 0.325† | −0.815** |
|  | (0.333) | (0.188) | (0.310) |
| Long-term illness | −2.289*** | 1.025*** | −5.557*** |
|  | (0.151) | (0.078) | (0.165) |
| Postgraduate degree | −1.262† | 0.337 | 2.416*** |
|  | (0.736) | (0.343) | (0.723) |
| Graduate diploma/certificate | −2.851*** | 1.145** | 1.257 |
|  | (0.772) | (0.405) | (0.808) |
| Bachelor or honours degree | −1.441** | 0.655** | 1.983*** |
|  | (0.499) | (0.254) | (0.505) |
| Advanced diploma/diploma | −0.858 | 0.457 | 2.123** |
|  | (0.643) | (0.336) | (0.696) |
| Certificate III or IV | −0.261 | 0.475* | 1.122* |
|  | (0.477) | (0.241) | (0.447) |
| Year 12 | −1.010** | 0.543** | −0.478 |
|  | (0.364) | (0.188) | (0.369) |
| Individual fixed effects | Yes | Yes | Yes |
| Survey-wave fixed effects | Yes | Yes | Yes |
| Observations | 110,639 | 55,132 | 109,971 |
| Individuals | 13,781 | 12,775 | 13,772 |
| Within R² | 0.014 | 0.024 | 0.051 |
| *Notes:* Cluster-robust standard errors at the individual level are reported in parentheses. All models include individual and survey-wave fixed effects and contemporaneous long-term illness. The reference category for educational attainment is Year 11 and below. Education coefficients are identified from within-woman changes in recorded educational attainment and should not be interpreted as conventional cross-sectional differences between education groups. Higher MHI-5 and SF-36 GH scores indicate better health, whereas higher K10 scores indicate greater psychological distress. † $p<0.10$; * $p<0.05$; ** $p<0.01$; *** $p<0.001$ | | | |

| **Appendix Table A6. Fixed-effects models additionally adjusting for labour-force status** | | | |
| --- | --- | --- | --- |
| **Variable** | **MHI-5** | **K10** | **General health** |
| Weather-related disaster exposure | −0.856* | 0.328† | −0.828** |
|  | (0.334) | (0.188) | (0.310) |
| Long-term illness | −2.276*** | 1.014*** | −5.548*** |
|  | (0.150) | (0.077) | (0.165) |
| Unemployed | −1.025*** | 0.624** | 0.263 |
|  | (0.304) | (0.190) | (0.265) |
| Not in the labour force | −0.145 | 0.050 | −0.550*** |
|  | (0.168) | (0.087) | (0.163) |
| Individual fixed effects | Yes | Yes | Yes |
| Survey-wave fixed effects | Yes | Yes | Yes |
| Observations | 110,678 | 55,153 | 110,009 |
| Individuals | 13,789 | 12,783 | 13,780 |
| Within R² | 0.014 | 0.024 | 0.051 |
| *Notes:* Cluster-robust standard errors at the individual level are reported in parentheses. All models include individual and survey-wave fixed effects and contemporaneous long-term illness. The reference category for labour-force status is employed. Labour-force status may change following disaster exposure or changes in health; these models are therefore treated as supplementary sensitivity analyses rather than primary confounder-adjusted specifications. Higher MHI-5 and SF-36 GH scores indicate better health, whereas higher K10 scores indicate greater psychological distress. † $p<0.10$; * $p<0.05$; ** $p<0.01$; *** $p<0.001$. | | | |

| **Appendix Table A7. Wave-by-wave next-wave retention by disaster-exposure status** | | | | |
| --- | --- | --- | --- | --- |
| **Wave transition** | **Non-exposed: retained/current (%)** | **Exposed: retained/current (%)** | **Adjusted difference, percentage points** | **p-value** |
| Wave 9 → 10 | 5,689/5,978 (95.17%) | 75/80 (93.75%) | −1.42 | 0.603 |
| Wave 10 → 11 | 5,911/6,215 (95.11%) | 103/110 (93.64%) | −1.47 | 0.530 |
| Wave 11 → 12 | 7,437/7,825 (95.04%) | 226/239 (94.56%) | −0.48 | 0.746 |
| Wave 12 → 13 | 7,611/8,026 (94.83%) | 119/123 (96.75%) | 1.92 | 0.236 |
| Wave 13 → 14 | 7,568/8,000 (94.60%) | 95/103 (92.23%) | −2.37 | 0.372 |
| Wave 14 → 15 | 7,765/8,126 (95.56%) | 54/59 (91.53%) | −4.03 | 0.267 |
| Wave 15 → 16 | 7,687/8,021 (95.84%) | 133/138 (96.38%) | 0.54 | 0.736 |
| Wave 16 → 17 | 8,035/8,438 (95.22%) | 91/98 (92.86%) | −2.37 | 0.365 |
| Wave 17 → 18 | 7,955/8,380 (94.93%) | 118/125 (94.40%) | −0.53 | 0.799 |
| Wave 18 → 19 | 7,893/8,260 (95.56%) | 72/74 (97.30%) | 1.74 | 0.359 |
| Wave 19 → 20 | 7,879/8,354 (94.31%) | 103/105 (98.10%) | 3.78 | 0.005 |
| Wave 20 → 21 | 7,757/8,252 (94.00%) | 97/104 (93.27%) | −0.73 | 0.767 |
| Wave 21 → 22 | 7,465/8,008 (93.22%) | 104/111 (93.69%) | 0.47 | 0.838 |
| **Overall** | **96,652/101,883 (94.87%)** | **1,390/1,469 (94.62%)** | **−0.24** | **0.683** |
| *Notes:* Retention indicates that a woman observed in the current survey wave was also observed in the immediately following wave. The analysis includes women aged 15 years and older with valid disaster-exposure information in the current wave. Percentages in the non-exposed and exposed columns are unadjusted retention rates. Wave-specific differences are average marginal effects from a logistic regression containing interactions between disaster-exposure status and survey wave, with cluster-robust standard errors at the individual level. Positive differences indicate higher retention among exposed observations. The overall adjusted difference was −0.24 percentage points (95% CI: −1.41 to 0.92; *p* = 0.683). A joint test found no evidence that exposure-related retention differences varied systematically across waves, $\chi^{2}(12)=8.66$, *p* = 0.732. Although the wave 19–20 comparison was statistically significant, the overall and joint tests did not indicate systematic differential attrition. | | | | |

**Appendix Figure A1. Callaway–Sant’Anna event-study estimates among women aged 18 years and older**

*Notes:* The analysis is restricted to women aged 18 years and older with valid disaster-exposure and outcome information who reported disaster exposure in no more than one survey wave. Women with no observed disaster exposure constitute the comparison group. Points represent event-time estimates, and vertical bars represent 95% confidence intervals. Panel A presents MHI-5 mental health, Panel B presents SF-36 general health, and Panel C presents K10 psychological distress. The general-health global pre-trend test was statistically significant (p = 0.037); that panel should therefore be interpreted cautiously.
