# Supplementary material for: Disaster exposure and women’s mental and general health: evidence from longitudinal data
Source: Glob Health Action. 2026 Oct 27;19(1):2752483. doi: 10.1080/16549716.2026.2752483 (PMC13715346; doi:10.1080/16549716.2026.2752483)
Supplement: Supplementary file 2 [file ZGHA_A_2752483_SM6030.docx]

**Supplementary file #2**

STROBE Statement—Checklist of items that should be included in reports of ***cohort studies***

**Title: Disaster Exposure and Women’s Mental and General Health: Evidence from Longitudinal Data**

|  | Item No | Recommendation |
| --- | --- | --- |
| **Title and abstract** | 1 | (*a*) The title identifies the study as a longitudinal study: *Disaster Exposure and Women's Mental and General Health: Evidence from Longitudinal Data.*. |
|  |  | (*b*) The abstract provides a balanced summary of the study rationale, nationally representative longitudinal HILDA Survey data, disaster exposure, health outcomes (mental health, self-rated general health, and psychological distress), statistical methods (fixed-effects panel models and event-study analysis), principal findings, and the public health implications of the results. |
| Introduction | | |
| Background/rationale | 2 | The Introduction describes the increasing frequency and severity of disasters and their implications for population health. It highlights the limited longitudinal evidence on disaster-related health outcomes, particularly for women, identifies the limitations of previous cross-sectional studies in addressing unobserved individual heterogeneity, and explains the rationale for using nationally representative longitudinal data and fixed-effects models to estimate the association between disaster exposure and women's mental and general health. |
| Objectives | 3 | The study objectives are clearly stated: to examine the association between disaster exposure and women's mental health, psychological distress, and self-rated general health using nationally representative longitudinal data; to estimate within-individual changes using fixed-effects panel models; and to assess the timing and persistence of health effects following disaster exposure using an event-study framework. |
| Methods | | |
| Study design | 4 | This study is an observational longitudinal analysis using secondary data from the Household, Income and Labour Dynamics in Australia (HILDA) Survey. The study employs fixed-effects panel models and an event-study framework to examine the association between disaster exposure and women's mental health, psychological distress, and self-rated general health. |
| Setting | 5 | The study setting is Australia. The analysis uses nationally representative longitudinal household survey data from the Household, Income and Labour Dynamics in Australia (HILDA) Survey, collected annually from 2001 to 2022, with repeated observations of participants across survey waves. |
| Participants | 6 | (*a*) Eligible participants were women in the HILDA Survey with valid information on disaster exposure and at least one valid health outcome measure, including mental health, psychological distress, or self-rated general health. Participants contributed repeated observations across survey waves from 2001 to 2022. |
|  |  | (*b*) Not applicable; this study did not use matching. |
| Variables | 7 | Disaster exposure was the primary exposure. Health outcomes included mental health (MHI-5), psychological distress (Kessler-6), and self-rated general health (SF-36 General Health). Covariates included age, long-term illness, and socioeconomic characteristics. Individual fixed effects controlled for all time-invariant observed and unobserved characteristics, and survey wave fixed effects accounted for common time trends. |
| Data sources/ measurement | 8* | Disaster exposure and all health outcome measures were obtained from the Household, Income and Labour Dynamics in Australia (HILDA) Survey. Mental health was measured using the Mental Health Inventory-5 (MHI-5), psychological distress using the Kessler-6 (K6) scale, and self-rated general health using the SF-36 General Health measure. Covariates were derived from standard HILDA Survey instruments and were measured consistently across survey waves. |
| Bias | 9 | Potential sources of bias were addressed through the use of validated HILDA Survey instruments, individual fixed-effects models to control for time-invariant observed and unobserved characteristics, survey wave fixed effects to account for common temporal trends, adjustment for time-varying covariates (including age, long-term illness, and socioeconomic characteristics), and robust standard errors clustered at the individual level. Residual confounding from unmeasured time-varying factors cannot be completely excluded. |
| Study size | 10 | The study size was determined by the number of women participating in the HILDA Survey who met the inclusion criteria and had valid information on disaster exposure, health outcomes, and covariates across the study period (2001–2022). |
| Quantitative variables | 11 | Disaster exposure was analysed as a binary variable. Mental health (MHI-5), psychological distress (Kessler-6), self-rated general health (SF-36 General Health), age, and other covariates were analysed in their original scales. Event time was modelled as a series of indicator variables representing survey waves before and after disaster exposure in the event-study analysis. |
| Statistical methods | 12 | (*a*) Associations between disaster exposure and women's health outcomes were estimated using individual fixed-effects panel regression models with survey wave fixed effects. Robust standard errors were clustered at the individual level to account for repeated observations. An event-study framework was also used to examine the timing and persistence of health changes before and after disaster exposure. |
|  |  | (*b*) Not applicable. No subgroup or interaction analyses were undertaken. |
|  |  | (*c*) The analytical sample was restricted to women with non-missing information on disaster exposure and the relevant health outcome. Complete-case analyses were undertaken for each model, and sample sizes differed across outcomes because of item non-response. |
|  |  | (*d*) The HILDA Survey is an unbalanced longitudinal panel in which participants contribute varying numbers of observations across survey waves. Individual fixed-effects models use all available observations for each participant, and no additional modelling of attrition was undertaken. |
|  |  | (*e*) Sensitivity analyses examined the robustness of the findings by estimating extended fixed-effects models that additionally adjusted for geographic remoteness, educational attainment, and employment status. Event-study analyses were also undertaken to assess the temporal dynamics of the estimated associations. |
| Results | | |
| Participants | 13* | (a) The numbers of observations and participants included in each analysis are reported for each outcome in the Results and regression tables. The analytical sample comprised women with valid information on disaster exposure and the relevant health outcome measures. Sample sizes differed across outcomes because of outcome availability and item non-response. |
|  |  | (b) The analytical sample was restricted to women with non-missing information on disaster exposure, the relevant health outcome, and covariates. Sample sizes varied across analyses because of item non-response and outcome availability. |
|  |  | (c) A flow diagram was not included because this study used secondary longitudinal survey data from an established national panel, with participants contributing repeated observations across survey waves. |
| Descriptive data | 14* | (a) Descriptive characteristics of the analytical sample are presented, including women's mental health (MHI-5), psychological distress (Kessler), self-rated general health (SF-36 General Health), age, long-term illness, and disaster exposure. Additional descriptive information on education, employment status, and geographic remoteness is presented in the Supplementary Appendix. |
|  |  | (b) The number of observations included for each variable and the sample sizes for each regression model are reported. Sample sizes differ across outcomes because of item non-response and outcome availability. |
|  |  | (c) Participants contributed repeated observations across multiple survey waves in the HILDA Survey. The panel was unbalanced, with individuals contributing varying numbers of observations over the study period. |
| Outcome data | 15* | Summary statistics for women's mental health (MHI-5), psychological distress (Kessler), and self-rated general health (SF-36 General Health) are presented in the descriptive statistics table. Model-specific sample sizes and outcome estimates are reported for each fixed-effects regression analysis. |
| Main results | 16 | (*a*) Unadjusted (baseline) and adjusted fixed-effects regression estimates are presented as regression coefficients with robust standard errors. The adjusted models control for age and long-term illness, while additional models examining geographic remoteness, educational attainment, and employment status are presented in the Supplementary Appendix. |
|  |  | (*b*) Not applicable. Continuous outcome variables were analysed in their original scales without categorisation. |
|  |  | (*c*) Not applicable. The study analysed continuous health outcomes using fixed-effects linear regression models and did not estimate relative risks or absolute risks. |
| Other analyses | 17 | Additional analyses included event-study models to examine the timing and persistence of health changes following disaster exposure and extended fixed-effects models that additionally adjusted for geographic remoteness, educational attainment, and employment status to assess the robustness of the findings. |
| Discussion | | |
| Key results | 18 | The key findings are summarised in relation to the study objectives. Disaster exposure was associated with significant declines in women's mental health and self-rated general health and increased psychological distress. The event-study analysis further demonstrated that these adverse health effects emerged around the time of disaster exposure and persisted over subsequent survey waves. |
| Limitations | 19 | Study limitations are discussed, including the use of self-reported disaster exposure, the possibility of residual confounding from unmeasured time-varying factors, the focus on adult women, the inability to examine heterogeneity by disaster type or exposure intensity, and the absence of direct measures of disaster severity. |
| Interpretation | 20 | The findings are interpreted cautiously in light of the longitudinal study design, existing literature, and study limitations. The Discussion considers the implications for women's health, health economics, disaster preparedness, health system planning, mental health service provision, and public policy. |
| Generalisability | 21 | The findings are generalisable to adult women in the Australian community-dwelling population represented by the nationally representative HILDA Survey. Generalisability to other populations and disaster contexts may be limited. |
| Other information | | |
| Funding | 22 | No funding was received for conducting this study |

*Give information separately for exposed and unexposed groups.

**Note:** An Explanation and Elaboration article discusses each checklist item and gives methodological background and published examples of transparent reporting. The STROBE checklist is best used in conjunction with this article (freely available on the Web sites of PLoS Medicine at http://www.plosmedicine.org/, Annals of Internal Medicine at http://www.annals.org/, and Epidemiology at http://www.epidem.com/). Information on the STROBE Initiative is available at http://www.strobe-statement.org.
